# Relationship between perceived body weight and body mass index based on self- reported height and weight among university students: a cross-sectional study in seven European countries

**DOI:** 10.1186/1471-2458-10-40

**Published:** 2010-01-27

**Authors:** Rafael T Mikolajczyk, Annette E Maxwell, Walid El Ansari, Christiane Stock, Janina Petkeviciene, Francisco Guillen-Grima

**Affiliations:** 1 Department of Public Health Medicine, School of Public Health, University of Bielefeld, Bielefeld, Germany; 2 Department of Clinical Epidemiology, Bremen Institute for Prevention Research and Social Medicine, Bremen, Germany; 3 School of Public Health and Jonsson Comprehensive Cancer Center, 650 Charles Young Dr. South, Los Angeles, CA 90095-6900, University of California, Los Angeles, USA; 4 Faculty of Sport, Health & Social Care, University of Gloucestershire, Gloucester, UK; 5 Unit for Health Promotion Research, University of Southern Denmark, Esbjerg, Denmark; 6 Preventive Medicine Department, Institute for Biomedical Research, Kaunas University of Medicine, Kaunas, Lithuania; 7 Department of Health Sciences, Public University of Navarra, Pamplona, Spain; 8 Department of Preventive Medicine and Public Health, University Clinic of Navarra, Pamplona, Spain

## Abstract

**Background:**

Despite low rates of obesity, many university students perceive themselves as overweight, especially women. This is of concern, because inappropriate weight perceptions can lead to unhealthy behaviours including eating disorders.

**Methods:**

We used the database from the Cross National Student Health Survey (CNSHS), consisting of 5,900 records of university students from Bulgaria, Denmark, Germany, Lithuania, Poland, Spain and Turkey to analyse differences in perceived weight status based on the question: "Do you consider yourself much too thin, a little too thin, just right, a little too fat or much too fat?". The association between perceived weight and body mass index (BMI) calculated from self-reported weight and height was assessed with generalized non-parametric regression in R library *gam*.

**Results:**

Although the majority of students reported a normal BMI (72-84% of males, 65-83% of females), only 32% to 68% of students considered their weight "just right". Around 20% of females with BMI of 20 kg/m^2 ^considered themselves "a little too fat" or "too fat", and the percentages increased to 60% for a BMI of 22.5 kg/m^2^. Male students rarely felt "a little too fat" or "too fat" below BMI of 22.5 kg/m^2^, but most felt too thin with a BMI of 20 kg/m^2^.

**Conclusions:**

Weight ideals are rather uniform across the European countries, with female students being more likely to perceive themselves as "too fat" at a normal BMI, while male students being more likely to perceive themselves as "too thin". Programs to prevent unhealthy behaviours to achieve ill-advised weight ideals may benefit students.

## Background

The growing rate of obesity in children and adults is a global health concern. In European countries, about 20% of children and adolescents and 30 to 80% of adults are overweight or obese with rising secular trends. High levels of overweight affect both Eastern and Western European countries [1,2]. Corresponding to this trend, large proportions of the population are unsatisfied with their weight and trying to lose weight. In addition to actual weight, *perceived *weight status is an important determinant of eating and weight-loss behaviour [3-7].

Perceived weight does not always reflect actual weight status based on body mass index (BMI). Studies have shown that despite low rates of obesity, many university students, especially women, perceive themselves as overweight [5,8-11]. This is of concern, because inappropriate weight perceptions can lead to unhealthy behaviours including eating disorders [5,12,13]. Universities and colleges, on the other hand, represent an opportunity for reaching a large number of students to promote appropriate weight perceptions and healthy eating behaviours [13].

Actual weight and weight perceptions may be influenced by food habits and food environments, nutritional knowledge, cultural norms and expectations and mass media depictions of what constitutes an ideal figure, in addition to lifestyle differences that affect physical activity. These factors may differ between Western and Eastern European countries [5,8,9]. However, few studies have examined weight and weight perceptions across various European countries. The Health Behaviour in School-Aged Children (HBSC) study, a cross-national survey conducted by the World Health Organization since 1982, has provided information on self-reported weight and weight perceptions among school-aged children [14,15]. This study found considerable variation across the studied countries in the prevalence of overweight, trying to lose weight and perceived need to lose weight [14]. The between-country variation in perceived need to lose weight was not only due to the different prevalences of overweight in participating countries, but also due to between-country variations in perceptions among overweight respondents. For example, overweight boys compared to non-overweight boys were almost 11 times more likely to try to lose weight in Denmark but only 3 times more likely in Russia. This analysis was limited to 11-, 13- and 15-year old school children. Even within this relatively narrow age range, body weight and weight perceptions changed significantly with age [14]. While HBSC considerably added to the knowledge of weight and weight perceptions among school age children across different countries, less is known about older adolescents and young adults. The International Health and Behaviour Survey (IHBS), which was conducted in 22 countries, reported perceived weight and BMI calculated from self-reportef height and weight for university students [8]. However, since the samples from participating countries were too small for separate analyses, the results were reported for five regions: Western European countries and the U.S., and Eastern European, Mediterranean, Pacific Asian and South American countries. Our study adds to this literature by providing weight perceptions and self-reported height and weight in student samples from seven European countries with sample sizes large enough for a country-specific analysis.

The aim of this analysis was to compare the relationship between perceived body weight and BMI based on self-reported height and weight in student populations across different European countries, including one Northern European country (Denmark), two Western European countries (Germany, Spain), three Eastern European countries (Poland, Bulgaria, Lithuania) and one South-Eastern European country (Turkey). Of particular relevance are countries that have recently joined the European Union (Bulgaria, 2007, Poland and Lithuania, 2004) and the candidate country Turkey, as these countries are currently undergoing economic and societal transitions with current and projected changes in economic growth. The expected economic development and growing Western European influence may lead to lifestyle changes that may also affect body weight and perceived body weight among student populations in these countries [9].

## Methods

### Sample and variables

We used the database from the Cross National Student Health Survey (CNSHS), consisting of 5,900 records of university students from seven countries in Europe [16]. The participating universities were: the University of Bielefeld, Germany (DE), the University of Lublin, Poland (PL), the University of Sofia, Bulgaria (BG), the Navarra Public University and the University of Navarra, Spain (ES), the Universities of Kaunas, Lithuania (LT), the University of Southern Denmark (DK) and Hacettepe University, Turkey (TR). For ease we use country names in the text and country codes in the tables. Data from four countries were collected between 2003 and 2005, while data from Spain and Lithuania were collected between 1998 and 2000. In Spain, students were recruited through an advertisement at the university campus, and about 28% of all first-year students at that university participated in this study. In all other countries, first-year students from randomly selected courses were invited to complete a self-administered questionnaire during the last 10 minutes of their lectures. Response rates in these countries ranged from 85% to 97%. Participation was voluntary and anonymous. Permission to conduct the study was obtained from the participating institutions. Students were informed that by filling out the questionnaire they were providing informed consent for the participation in the study. They were also instructed that they can withdraw from the study at any point by not returning the questionnaires. In Spain, where additional biomedical measurements were conducted and blood samples were obtained, the study was approved by the local ethics committee, and written informed consent was obtained from all participants.

Among the 5,900 participants, 558 (9%) did not report either their weight or height. The fraction of missing responses was substantially higher in Spain (32%) than in other countries (2-8%). This is probably resulting from the fact that students in Spain completed the questionnaire before having physical exams including weight and height measurement and either did not know their exact weight and/or height or did not feel that it was necessary to report the information. Students with missing information on weight or height did not differ with respect to measured weight from students who self-reported weight and height. Because weight and height were only measured in Spain, this analysis is based on self-reports and restricted to students who reported weight and height. In the Spanish sample, students on average underreported weight by 0.60 kg and overreported height by 1.07 cm (unpublished data). This resulted in an average underreporting of BMI of -0.49 kg/m^2^.

The number of respondents in each country and their gender and age distributions are presented in Table 1. The proportion of female students was greater than 50% in all countries except Denmark. Students from Poland, Bulgaria and Spain were the youngest, whereas in Germany and Denmark, a considerable proportion of students were older than 23 years.

**Table 1:** Demographic and self-reported anthropometrical characteristics of students in 7 European countries

Characteristics	DE, 2005 N = 739	DK, 2005 N = 530	PL, 2005 N = 564	BG, 2005 N = 692	TR, 2003 N = 1005	LT, 2000 N = 1016	ES, 1998 N = 796
%							
Gender							
Female	56.8	48.1	71.1	68.3	69.5	54.0	63.0
Male	43.2	51.9	28.9	31.7	30.5	46.0	37.0
Age (years)							
<20	1.8	5.3	22.7	54.7	49.8	56.9	75.8
20-23	78.6	67.4	76.4	43.2	46.0	40.2	22.3
>23	19.6	27.4	.9	2.0	4.3	2.9	1.9
mean (standard deviation)							
Height (cm)							
Female	169 (7)	168 (6)	166 (6)	167 (6)	165 (6)	171 (8)	165 (6)
Male	182 (8)	182 (7)	180 (9)	180 (7)	177 (7)	179 (8)	178 (7)
Weight (kg)							
Female	62 (10)	64 (11)	58 (10)	56 (9)	55 (7)	62 (10)	58 (8)
Male	79 (14)	79 (12)	73 (10)	73 (10)	71 (10)	71 (10)	73 (10)
BMI (kg/m^2^)							
Female	21.9 (4.2)	22.4 (3.7)	20.8 (3.3)	19.9 (2.6)	20.5 (2.4)	21.0 (2.3)	21.3 (2.8)
Male	23.8 (4.2)	23.8 (3.0)	22.6 (4.0)	22.4 (2.5)	22.5 (2.7)	22.1 (2.4)	23.1 (2.5)

In order to assess students' perceived weight, they were asked: "Do you consider yourself much too thin, a little too thin, just right, a little too fat or much too fat?" BMI was calculated from self-reported weight (in kilograms) and height (in centimetres).

### Statistical analysis

Because we relied on students' self-reported height and weight, we estimated the extent of guessing or rounding by examining the data for last digit preference, e.g., overreporting of numbers ending in zero or five [17]. We calculated the proportion of responses with last digit "0" or "5" by country and by gender. To assess whether the proportions differed between countries within each gender we used DerSimonian Laird estimator [18]. DerSimonian Laird estimator is a test for heterogeneity across different samples commonly used in meta-analyses; it is equivalent to testing whether the variance of random effects is different from zero. The test was conducted using software META^® ^[19]. Additionally, we studied the differences in last digit preferences across BMI categories in male and female students. Self-reported weight and height and calculated BMI were displayed as means for each country and gender. Given the sample sizes in different countries (stratified by gender), differences of ≥ 3 kg in weight, ≥ 2 cm in height and ≥ 1 kg/m^2 ^in BMI are statistically significant at p < 0.05 (statistical significance of pair-wise comparisons is not reported in the text). These differences also appear to be reasonably meaningful. In order to provide further information, we also estimated the range of effect sizes in terms of Cohen's d for differences between genders and across countries for weight, height and BMI.

According to WHO guidelines [20], students were classified as underweight (BMI < 18.5 kg/m^2^), normal weight (BMI of 18.5-24.9 kg/m^2^), overweight (BMI of 25.0-29.9 kg/m^2^), or obese (BMI ≥ 30.0 kg/m^2^), and the proportions of students in these categories were computed in each country separately for males and females. Given that BMI based on self-reported data is usually biased downwards [21], we conducted a sensitivity analysis increasing the reported weight by 1 kg in all participants, which is somewhat larger than the underreporting observed in Spain. This increase in weight resulted in an increase in BMI by 0.30 to 0.37 kg/m^2^, which is about twice as much as the underreporting bias seen in our own validation study conducted in Germany [22], and somewhat less than the estimate of underreporting in the Spanish students above.

Finally, we investigated how perceived body weight was related to the BMI reported by students. Three separate models with dichotomous responses were employed: 1) "just right" versus the remaining categories, 2) "much too thin" or "little too thin" versus the remaining categories, 3) "much too fat" and "little too fat" versus the remaining categories. The probability of a given response across the BMI spectrum was modelled using non-parametric regression with locally weighted polynomial fit (*loess *function, implemented in R library *gam *[23]. Non-parametric regression allows the assessment of the form of the association between the independent and dependent variables. The analysis was conducted separately for both genders and each country; the existence of statistical differences across these strata was tested using an interaction term in a joint model. Again, the analysis was repeated with BMI calculated from reported weight increased by 1 kg for all participants.

## Results

### Last digit preference for self-reported weight or height

**Figure 1:**
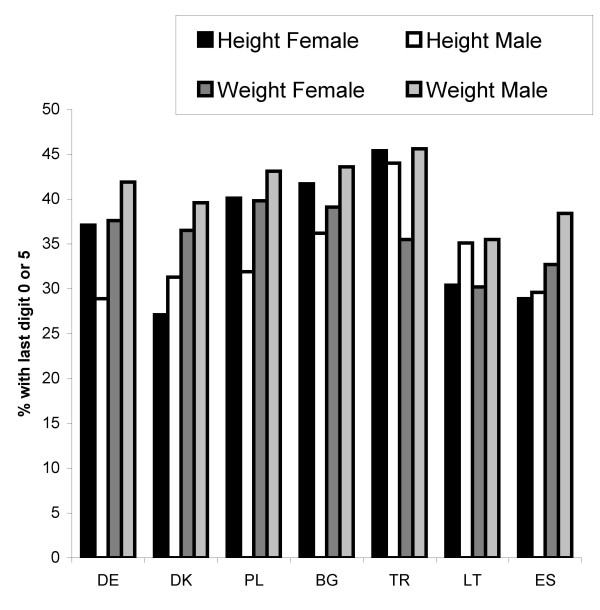
**Last digit preference for self-reported weight and height by country and gender**. Note: 95% confidence intervals for the proportions are ± 4-5% for female and ± 4-7% for male students in each of the countries.

**Figure 2:**
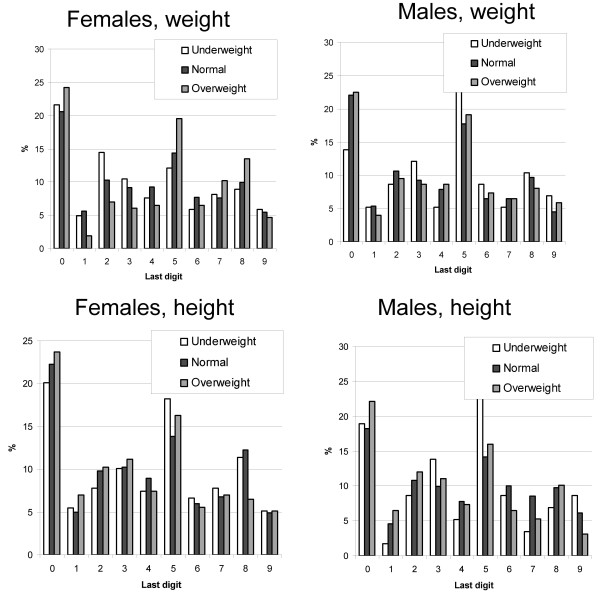
**Last digit preference for self-reported weight and height by gender and BMI category**.

There was a considerable preference for last digits 0 and 5 in self-reported weight or height, as in all cases the fraction of such reports considerably exceeded the expected 20% (Figure 1). There was also some variation between the countries and by gender in the extent of this preference (heterogeneity test p < 0.001), but in general, the extent of preference was rather similar. Female students had slightly lower last digit preference for weight than male students. The extent of last digit preference across both genders was lowest in Lithuania and highest in Turkey. The lowest levels of last digit preference were in height among male students in Germany and Spain. Additionally, we investigated whether the extent of last digit preference depended on BMI category based on self-reported weight and height. Since there was little variation across countries, we performed a pooled analysis across all countries. A clear difference appeared only in females and only with respect to weight: overweight femalgs were more likely to display a last figit preference than normal or underwekgjt females (Figure 2). Inspection of the last digit of eivher reported weight or height suggest that occurrence of rounding was most likely for digits "1" and "9" rounded to "0" and for digits "4" and "6" rounded to "5".

### Differences in height, weight and prevalence of overweight between countries and genders

Country differences in height and weight were slightly more pronounced in female than in male students (partial eta-square among females 0.11 for height and 0.10 for weight, among males 0.06 for height and 0.09 for weight). Female students were shorter in Southern (Spain, Turkey) and Eastern European (Poland, Bulgaria) countries than in Western or Northern European countries (Germany, Denmark, and Lithuania) (see Table 1). In terms of Cohen's effect sizes, the pairwise differences within the above groups (Southern, Eastern etc.) were in most cases below d = 0.2, which means small effects; in contrast differences between countries from discordant groups were d > 0.2. Lithuanian women were particularly tall, with a difference of 6 cm in height to either Spanish or Turkish female students (effect size of 0.85). The pattern was similar for the height of male students, with the exception of Lithuania. Since males from Lithuania tended to be shorter, the difference between both genders was exceptionally small in Lithuania.

There were also some differences with respect to weight: female and male students had the highest mean weight in Germany and Denmark. In the case of weight, the above defined grouping of countries was less demarked, with somewhat larger differences within groups (difference in mean weight between females from Turkey and Spain of 3 kg; d = 0.4), but still substantial differences across the spectrum of all countries: maximum difference of 8 kg across countries, d ≥ 0.7. Difference in weight by gender was smallest in Lithuania (d = 0.9) and largest in Bulgaria (d = 1.8). Evaluation of BMI based on self-reported weight and height showed that the differences between countries were somewhat smaller for male students (maximum difference of 1.7 kg/m^2^, d = 0.63) and somewhat larger for female students (maximum difference of 2.5 kg/m^2^, d = 0.78) with an exceptionally low BMI in female Bulgarian students.

Based on self-reported weight and height, 65% to 83% of the female students had a BMI in the normal category (Table 2). Overweight and obesity were most prevalent in Danish females followed by German females. Conversely, underweight was highly prevalent in both Slavic countries (Poland and Bulgaria) and in Turkey, with particularly high values in Bulgaria. The patterns were similar for male students (72-84% BMI in the normal category), but males had a considerably higher prevalence of overweight and a much lower prevalence of underweight than females. For both genders, the highest proportion of students with normal weight was in Lithuania. In the sensitivity analysis using increased weight in all participants, the only substantial difference was related to the fraction of underweight females, which was lower by up to five percentage points in Poland, Bulgaria and Turkey (data not shown), than in Table 2. Nevertheless, the corresponding fractions remained high in these countries even after the correction.

**Table 2:** BMI categories and perceived weight status by country and gender (%)

Characteristics	DE, 2005 N = 739	DK, 2005 N = 530	PL, 2005 N = 564	BG, 2005 N = 692	TR, 2003 N = 1005	LT, 2000 N = 1016	ES, 1998 N = 796
BMI Categories (derived from self-reported height and weight)							
Female							
Underweight	9.2	6.7	16.8	30.4	20.8	11.5	11.0
Normal weight	78.8	75.3	76.1	64.9	74.2	83.4	79.4
Overweight	8.9	13.3	5.8	4.0	4.7	4.7	8.6
Obese	3.1	4.7	1.3	.6	.3	.4	1.0
Male							
Underweight	1.0	1.5	2.5	5.0	4.2	3.9	1.7
Normal weight	71.7	74.2	81.9	81.2	80.1	84.2	80.6
Overweight	22.2	20.0	14.4	12.8	14.7	11.8	16.7
Obese	5.1	4.4	1.3	.9	1.0	.2	1.0
Perceived weight status							
Female							
Much too thin	.5		.8	1.1	1.6	.9	4.3
A little too thin	3.7	5.1	6.4	5.6	17.7	8.4	9.1
Just right	38.2	44.3	38.6	47.2	45.4	67.9	44.5
A little too fat	51.0	44.3	47.3	44.2	34.0	22.1	38.7
Much too fat	6.6	6.3	6.9	1.9	1.3	.7	3.4
Male							
Much too thin	3.8	1.1	8.3	3.7	2.0	3.4	4.5
A little too thin	21.3	13.1	29.5	22.9	25.8	20.5	19.4
Just right	32.1	53.8	39.7	52.3	50.2	63.8	48.8
A little too fat	37.8	29.5	19.9	20.2	21.7	11.4	25.3
Much too fat	5.1	2.5	2.6	.9	.3	.9	2.1

### Differences in perceived weight

Although the findings indicated a high proportion of normal weight among the sample, only 32% to 68% of students considered their weight "just right" (Table 2). Between 22% (Turkey) and 51% (Germany) of female students considered themselves "a little too fat". Only in Turkey was there a substantial fraction of female students who considered themselves too thin. Among males, between 11% (Lithuania) and 38% (Germany) of the sample considered themselves "a little too fat", with substantial proportions in all countries who considered themselves "a little to thin" (13% in Denmark, 30% in Poland).

**Figure 3:**
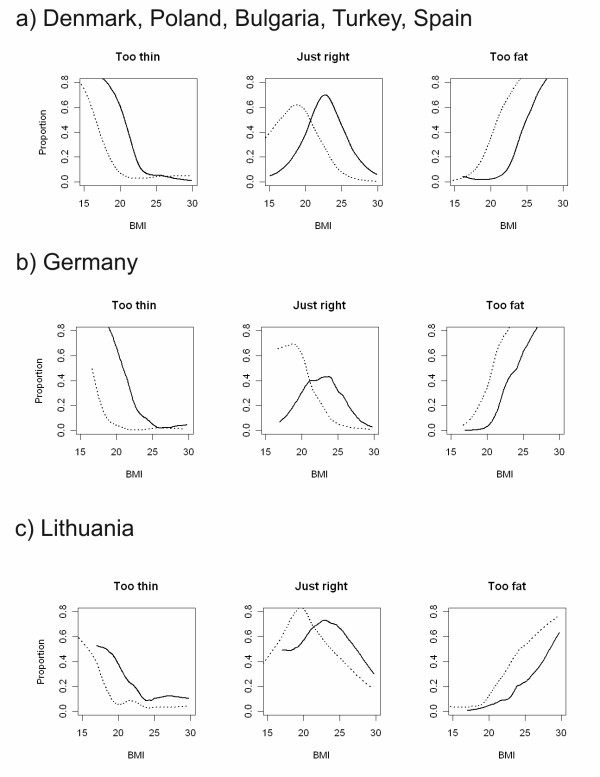
**Proportion of respondents with different BMI considering their weight just right, too fat and too thin by gender**. (dotted line - female, solid line - male, restricted to BMI < 30 kg/m^2^)

While Table 2 simply compared the distributions of BMI and perceived weight status by gender and country, the discrepancies between BMI and perceived weight status and the gender differences become even more apparent when they are considered jointly. In Figure 3, we combined students from Denmark, Poland, Bulgaria, Turkey and Spain because their country plots were very similar. We depicted Germany and Lithuania separately because they deviated from the general pattern.

In the first panel of Figure 3, less than 70% of students considered their weight "just right" for any given BMI. More significantly, female students felt "just right" at a lower BMI than their male peers did. Around 20% of females with BMI of 20 kg/m^2 ^considered themselves "too fat", and the percentages increased to 60% for a BMI of 22.5 kg/m^2^. Male students rarely felt "too fat" below BMI of 22.5 kg/m^2^. Conversely, most male students felt too thin with a BMI of 20 kg/m^2^.

In the German sample, only about 40% of male students considered their weight "just right", a much smaller proportion than in all other countries. In Lithuania, the proportion of women considering their weight "just right" remained reasonably high for normal BMI. However, this was also accompanied in Lithuania by a relatively high proportion of female students with BMI above 25 kg/m still considering their weight "just right".

## Discussion

Male students generally reported a higher BMI than female students, and there was a tendency toward lower BMIs in the Eastern European countries Poland, Bulgaria and Lithuania as compared to the Southern and Western European countries Germany, Denmark and Spain. Samples from the different countries differed substantially with respect to their BMI category distributions. While between 10% and 18% of females and between 18% and 27% of males in the Western and Southern European countries were overweight or obese, the prevalence was substantially lower in the Eastern European countries and in Turkey. We also found large differences with respect to the proportions of students who perceived themselves to be "a little" or "much too fat". Female students in all countries were more likely to describe themselves in this way than male students. Females in Germany, Denmark, and Poland, and males in Germany and Denmark were most likely to describe themselves as overweight. The substantial variation across countries is consistent with findings from the study comparing university students from 22 countries [8] and with cross-national studies in younger adolescents [24,25]. For countries that were included in IHBS as well as in our study (Poland, Germany, Bulgaria and Spain), the self-reported weight, height and BMI agreed very well [8]. For Lithuania, a 2002 study using measured weight and height reported data for 18-year-old boys and girls matching our findings very well[26].

When we considered BMI calculated from self-reported data and perceived weight jointly, we found a substantial level of misperceptions regarding body weight in students from European countries. These perceptions regarding body weight as related to BMI consistently differed by gender but were similar across countries. Most female students described their weight as "just right" at a BMI <20 kg/m^2^, which is in the low range of normal BMI, whereas most male students described their weight as "just right" at a BMI around 24 kg/m^2^, which is in the upper range of normal BMI. In the sensitivity analysis, all corresponding lines were shifted by approximately 0.3 kg/m^2 ^to the right - leaving all findings described above virtually unchanged. Our analysis differs from the previous analysis conducted by Wardle et al. [8] who used country-standardised deciles of BMI as the independent variable related to perceived weight in order to avoid using a BMI cut-point. We avoided this problem by treating BMI as a continuous variable in our analysis of association between reported BMI and perceived weight status.

Among Lithuanian students, we observed similar gender differences but an overall higher proportion of students rated their weight as "just right" over a wider range of BMI scores. In Lithuania there were substantially smaller differences in terms of weight, height and BMI between genders than in the other countries; the small differences might result from lower importance of body shape as a measure of attractiveness and subsequently in a less sharp delineation of ideal body weight. While we did not assess ideals of attractiveness in our study, other studies have found that population groups differ in their ideals regarding body weight [25]. Among German male students, an exceptionally low proportion reported "being just right", and a large proportion considered themselves too fat. Future studies are necessary to confirm these findings and to examine potential explanation for the differences we found.

Apart from the above dissimilarities, our findings suggest that weight ideals are rather uniform across the European countries and different for male and female university students. A multinational study among school-age children displayed a substantial variation in perceived need or attempts to lose weight among overweight boys and girls across 36 countries [14]. The data are not directly comparable to ours, because only a joint overweight category was reported and differences in weight distribution within this category could introduce additional variation across countries. Nevertheless, assuming a typical skewed distribution of BMI, the results for overweight adolescents should be more similar across countries in the absence of country-specific effects. Therefore, the findings in school-age children (11-, 13- and 15-year olds) appear in some contrast to our findings in university students. One possible explanation is that at younger ages, perceptions are more likely to be shared among peers (and therefore country specific responses exist), while in early adulthood more homogenous perceptions are created by media. Future longitudinal studies are needed to assess this issue. Interestingly, data from the HBSC study also suggest that Lithuanian overweight adolescents are among those with lowest attempts or perceived need to lose weight [14], which is in agreement with our results.

Females are more likely to perceive a lean body as ideal, which may be fuelled by images of thin women portrayed in the media [27-29]. In a recent study among female college students in the United States, 39% of normal weight students named media as a source of pressure to be a certain weight [30]. This is a public health concern, because females who restrict their food intake in order to achieve or maintain their desired body weight may have a nutrient intake that is inadequate for optimal health and may develop eating disorders [4]. In the study by Wardle and colleagues [8], about 50% of all female students from 22 countries were trying to lose weight, although only 5% had a BMI ≥ 25 kg/m^2^. Because several studies have found high rates of problem eating behaviours in university students, prevention programs for high risk female students may be appropriate [13].

The preference of males for a slightly heavier build may be due to male ideals of being "muscular" [31,32] and the absence of positive media portrayals of extremely thin males. The fact that males were more likely than females to rate their weight as "just right" even at higher BMI suggests that they are less likely to perceive themselves as being overweight. Other researchers who studied university students had similar findings: Wardle et al. [8] showed that the proportion of males who were trying to lose weight was substantially lower than among females. A study by Mintz and Betz [27] among students in the United States showed that men tended to perceive themselves as normal if they were actually overweight. Studies are ongoing to further examine this drive for muscularity, especially among male students [28,33].

### Strengths and Limitations

As strength, this study included a relatively large number of students from several European countries. With the exception of Spain, students were not explicitly informed that the survey included questions about weight. Therefore, it is unlikely that students declined participation because of this aspect. Still, there may be a selection bias, because students not interested in the health survey may differ in respect to their weight and their weight perceptions from their peers who participated in the study. Due to the high response rates achieved, this bias seems to be limited in all countries except Spain, where the response rate was substantially lower. However, findings from Spain were well in line with other countries, suggesting that the selection bias related to self-reported weight and weight perception may be similar in all countries included in this analysis.

As in the International Health and Behaviour Survey and the Health Behaviour of School Aged Children studies, our measure of BMI was based on self-reported weight and height. The reliability and validity of self-reported weight and height in different populations has been controversially discussed [34-41]. Most studies found a limited underestimation of BMI but with partly substantial effects on prevalence estimates for BMI categories. A recent review of studies conducted among adolescents in the United States indicated mean differences in self-reported versus directly measured BMI of -2.3 to 0.2 in females and -3.0 to -0.1 in males. While differences between self-reported and directly measured BMI were very small in nationally representative surveys, they were substantially larger in convenience samples or locally-based surveys [21]. Among other variables, the differences between reported and measured BMI might depend on education and age. An underestimation of 0.98 kg/m^2 ^in females and 0.75 in males for BMI was found in Greek adolescents [38]. A recent analysis using German HBSC sample demonstrated that adolescents, especially younger adolescents often do not report their weight or height, possibly because they do not know them [42]. Large proportions of missing BMI data were found in HBSC samples from many countries as well [14]. University students could be considered as the population group with more accurate, maybe best possible reporting, given their high education status and age range in early adulthood. In our own validation study in a German sample of students, we found an acceptable agreement between self-reported and measured BMI (mean difference of 0.18 kg/m^2^) [22]. Depending on the distribution of BMI, in some countries the underestimation might affect the prevalence of some BMI categories more than in others, but as demonstrated in the sensitivity analysis, correcting for a homogenous underestimation of weight did not change the qualitative differences observed across countries. More complex assumptions about underreporting of weight and possibly overreporting of height could result in further changes, therefore our estimates of the prevalence of BMI categories should be treated with caution. There could be cultural differences in the accuracy of reported BMI, but our analysis on last digit preferences for self-reported height and weight does not support this notion. On the other hand, the underreporting would affect findings presented in Figure 3 only slightly, lending further support to studies that use self-reported BMI for correlational analyses (in contrast to the estimation of prevalence of overweight) [43,44].

As many other studies [4,8] have done, we assessed perceived weight using a single question. Future research should use more sophisticated instruments to assess body perceptions with regard to muscularity, height and body fat distribution [33].

Our data were generated from student samples at one or two universities per country and may not be representative of all students of the respective countries. A comparison with representative data on students' age and gender distribution [45] confirms that Danish and German students are on average older than students from the other countries that are included in this analysis, and that there are more female than male students. Nevertheless, any distortion of the gender distribution is not likely to affect our results as the analysis was stratified by gender. Finally, the study was not conducted in all countries in the same year and while we do not expect large changes during a five-year period, some changes might have occurred.

## Conclusions

In conclusion, we found gender differences with regard to perceived weight related to BMI that were consistent among students from seven European countries: at a normal self-reported BMI, female students were more likely than male students to perceive themselves as "too fat", while male students were more likely to perceive themselves as "too thin". Future studies should address the potentially conflicting ideals of lean or muscular body shape among male students and associated health behaviours. Universities may be excellent settings to address misperceptions and to influence norms regarding body weight in order to prevent unhealthy behaviours among students to achieve ill-advised weight ideals.

## Competing interests

The authors declare that they have no competing interests.

## Authors' contributions

RTM designed the research question, conducted the analysis and drafted the manuscript. AEM wrote the final manuscript. WE, CS, JP and FG participated in writing the manuscript. All authors have read and approved the final manuscript.

## Pre-publication history

The pre-publication history for this paper can be accessed here:

http://www.biomedcentral.com/1471-2458/10/40/prepub

## References

[B1] WHO, Regional Office for Europe The challenge of obesity in the WHO European Region and the strategies for response http://www.euro.who.int/document/E90711.pdf [accessed 2009 June 5]

[B2] Knai C Suhrcke M Lobstein T Obesity in Eastern Europe: an overview of its health and economic implications Econ Hum Biol 2007 10 392 408 10.1016/j.ehb.2007.08.002 17920000

[B3] Cheung P Ip PL Lam ST Bibby H A study on body weight perception and weight control behaviours among adolescents in Hong Kong Hong Kong Med J 2007 10 16 21 17277387

[B4] ter Bogt TF van Dorsselaer SA Monshouwer K Verdurmen JE Engels RC Vollebergh WA Body mass index and body weight perception as risk factors for internalizing and externalizing problem behavior among adolescents J Adolesc Health 2006 10 27 34 10.1016/j.jadohealth.2005.09.007 16781958

[B5] Bellisle F Monneuse MO Steptoe A Wardle J Weight concerns and eating patterns: a survey of university students in Europe Int J Obes Relat Metab Disord 1995 10 723 730 8589766

[B6] Wardle J Johnson F Weight and dieting: examining levels of weight concern in British adults Int J Obes Relat Metab Disord 2002 10 1144 1149 10.1038/sj.ijo.0802046 12119582

[B7] Blokstra A Burns CM Seidell JC Perception of weight status and dieting behaviour in Dutch men and women Int J Obes Relat Metab Disord 1999 10 7 17 10.1038/sj.ijo.0800803 10094580

[B8] Wardle J Haase AM Steptoe A Body image and weight control in young adults: international comparisons in university students from 22 countries Int J Obes (Lond) 2006 10 644 651 10.1038/sj.ijo.0803050 16151414

[B9] Stock C Kücük N Miseviciene I Petkeviciene J Krämer A Misperceptions of body weight among university students from Germany and Lithuania Health Education 2004 10 113 121 10.1108/09654280410525559

[B10] Monneuse MO Bellisle F Koppert G Eating habits food and health related attitudes and beliefs reported by French students Eur J Clin Nutr 1997 10 46 53 10.1038/sj.ejcn.1600361 9023467

[B11] Anstine D Grinenko D Rapid screening for disordered eating in college-aged females in the primary care setting J Adolesc Health 2000 10 338 342 10.1016/S1054-139X(99)00120-2 10775826

[B12] Uzun O Gulec N Ozsahin A Doruk A Ozdemir B Caliskan U Screening disordered eating attitudes and eating disorders in a sample of Turkish female college students Compr Psychiatry 2006 10 123 126 10.1016/j.comppsych.2005.05.004 16490570

[B13] Sepulveda AR Carrobles JA Gandarillas A Poveda J Pastor V Prevention program for disturbed eating and body dissatisfaction in a Spanish university population: a pilot study Body Image 2007 10 317 328 10.1016/j.bodyim.2007.05.001 18089278

[B14] Ojala K Vereecken C Valimaa R Currie C Villberg J Tynjala J Kannas L Attempts to lose weight among overweight and non-overweight adolescents: a cross-national survey Int J Behav Nutr Phys Act 2007 10 50 10.1186/1479-5868-4-50 17935629 PMC2174511

[B15] Health Behaviour in School-Aged Children http://www.hbsc.org [accessed 2009 June 5]

[B16] El Ansari W Maxwell AE Mikolajczyk RT Stock C Naydenova V Kraemer A Promoting Public Health: Benefits and Challenges of a Europeanwide Research Consortium on Student Health Cent Eur J Public Health 2007 10 58 65 17645218 10.21101/cejph.a3418

[B17] Al-Marzouki S Evans S Marshall T Roberts I Are these data real? Statistical methods for the detection of data fabrication in clinical trials Bmj 2005 10 267 270 10.1136/bmj.331.7511.267 16052019 PMC1181267

[B18] Bohning D Malzahn U Dietz E Schlattmann P Viwatwongkasem C Biggeri C Some general points in estimating heterogeneity variance with the DerSimonian-Laird estimator Biostatistics 2022 10 445 657 10.1093/biostatistics/3.4.445 12933591

[B19] Schlattmann P Malzahn U Böhning D Schulze R, Holling H, Böhning D META - A Software Package for Meta-Analysis Meta-Analysis New Developments and Applications in Medical and Social Sciences 2003 Cambridge: Hogrefe Publishing Corp 251 258

[B20] World Health Organization Obesity: preventing and managing the global epidemic Book Obesity: preventing and managing the global epidemic (Editor ed.^eds.) 2000 City: Geneva: World Health Organization 11234459

[B21] Sherry B Jefferds ME Grummer-Strawn LM Accuracy of adolescent self-report of height and weight in assessing overweight status: a literature review Arch Pediatr Adolesc Med 2007 10 1154 1161 10.1001/archpedi.161.12.1154 18056560

[B22] Stock C Wille L Kramer A Gender-specific health behaviors of German university students predict the interest in campus health promotion Health Promot Int 2001 10 145 154 10.1093/heapro/16.2.145 11356753

[B23] Hastie TJ Chambers JM, Hastie TJ Generalized additive models Statistical Models in S 1991 10 Wadsworth & Brooks/Cole

[B24] Lissau I Overweight and obesity epidemic among children. Answer from European countries Int J Obes Relat Metab Disord 2004 10 Suppl 3 S10 15 10.1038/sj.ijo.0802822 15543208

[B25] Strauss RS Comparison of measured and self-reported weight and height in a cross-sectional sample of young adolescents Knt J Obes Relat Metab Disord 1999 10 904 908 10.1038/sj.ijo.0800971 10490794

[B26] Tutkuviene J Body mass index prevalence of overweight and obesity in Lithuanian children and adolescents 1985 2002 Coll Antropol 2007 10 109 121 17598389

[B27] Mintz LB Betz NE Sex differences in the nature realism and correlates of body image Sex Roles 1986 10 185 195 10.1007/BF00287483

[B28] McCreary DR Sasse DK An exploration of the drive for muscularity in adolescent boys and girls J Am Coll Health 2000 10 297 304 10.1080/07448480009596271 10863873

[B29] Pope HG Jr Gruber AJ Mangweth B Bureau B deCol C Jouvent R Hudson JI Body image perception among men in three countries Am J Psychiatry 2000 10 1297 1301 10.1176/appi.ajp.157.8.1297 10910794

[B30] Malinauskas BM Raedeke TD Aeby VG Smith JL Dallas MB Dieting practices weight perceptions and body composition: a comparison of normal weight overweight and obese college females Nutr J 2006 10 11 10.1186/1475-2891-5-11 16579846 PMC1456978

[B31] Lorenzen LA Exposure to Muscular Male Models Decreases Men's Body Satisfaction Sex Roles 2004 10 743 748 10.1007/s11199-004-0723-0

[B32] Cohane GH Pope HG Jr Body image in boys: a review of the literature Int J Eat Disord 2001 10 373 379 10.1002/eat.1033 11285574

[B33] Bergeron D Tylka TL Support for the uniqueness of body dissatisfaction from drive for muscularity among men Body Image 2007 10 288 295 10.1016/j.bodyim.2007.05.002 18089275

[B34] Elgar FJ Roberts C Tudor-Smith C Moore L Validity of self-reported height and weight and predictors of bias in adolescents J Adolesc Health 2005 10 371 375 10.1016/j.jadohealth.2004.07.014 16227121

[B35] Villanueva EV The validity of self-reported weight in US adults: a population based cross-sectional study BMC Public Health 2001 10 11 10.1186/1471-2458-1-11 11716792 PMC59896

[B36] Kuczmarski MF Kuczmarski RJ Najjar M Effects of age on validity of self-reported height weight and body mass index: findings from the Third National Health and Nutrition Examination Survey 1988 1994 J Am Diet Assoc 2001 10 28 34 quiz 35-26 10.1016/S0002-8223(01)00008-6 11209581

[B37] Niedhammer I Bugel I Bonenfant S Goldberg M Leclerc A Validity of self-reported weight and height in the French GAZEL cohort Int J Obes Relat Metab Disord 2000 10 1111 1118 10.1038/sj.ijo.0801375 11033979

[B38] Tsigilis N Can secondary school students' self-reported measures of height and weight be trusted? An effect size approach Eur J Public Health 2006 10 532 535 10.1093/eurpub/ckl050 16601105

[B39] Gillum RF Sempos CT Ethnic variation in validity of classification of overweight and obesity using self-reported weight and height in American women and men: the Third National Health and Nutrition Examination Survey Nutr J 2005 10 27 10.1186/1475-2891-4-27 16209706 PMC1262765

[B40] Brener ND McManus T Galuska DA Lowry R Wechsler H Reliability and validity of self-reported height and weight among high school students J Adolesc Health 2003 10 281 287 10.1016/S1054-139X(02)00708-5 12667732

[B41] Tokmakidis SP Christodoulos AD Mantzouranis NI Validity of self-reported anthropometric values used to assess body mass index and estimate obesity in Greek school children J Adolesc Health 2007 10 305 310 10.1016/j.jadohealth.2006.10.001 17367722

[B42] Mikolajczyk RT Richter M Associations of behavioural psychosocial and socioeconomic factors with over- and underweight among German adolescents Int J Public Health 2008 10 214 220 10.1007/s00038-008-7123-0 18716726

[B43] Goodman E Hinden BR Khandelwal S Accuracy of teen and parental reports of obesity and body mass index Pediatrics 2000 10 52 58 10.1542/peds.106.1.52 10878149

[B44] Pietilainen KH Kaprio J Borg P Plasqui G Yki-Jarvinen H Kujala UM Rose RJ Westerterp KR Rissanen A Physical inactivity and obesity: a vicious circle Obesity (Silver Spring) 2008 10 409 414 10.1038/oby.2007.72 18239652 PMC2249563

[B45] Education and Culture DG Key data on higher education in Europe 2007 http://eacea.ec.europa.eu/ressources/eurydice/pdf/0_integral/088EN.pdf

